# Comparison of phylogenetic trees through alignment of embedded evolutionary distances

**DOI:** 10.1186/1471-2105-10-423

**Published:** 2009-12-15

**Authors:** Kwangbom Choi, Shawn M Gomez

**Affiliations:** 1 Curriculum in Bioinformatics and Computational Biology, University of North Carolina at Chapel Hill, Chapel Hill, North Carolina, USA; 2 Department of Computer Science, University of North Carolina at Chapel Hill, Chapel Hill, North Carolina, USA; 3 Department of Pharmacology, University of North Carolina School of Medicine, Chapel Hill, North Carolina, USA; 4 Joint Department of Biomedical Engineering, University of North Carolina School of Medicine, Chapel Hill, North Carolina, USA

## Abstract

**Background:**

The understanding of evolutionary relationships is a fundamental aspect of modern biology, with the phylogenetic tree being a primary tool for describing these associations. However, comparison of trees for the purpose of assessing similarity and the quantification of various biological processes remains a significant challenge.

**Results:**

We describe a novel approach for the comparison of phylogenetic distance information based on the alignment of representative high-dimensional embeddings (xCEED: Comparison of Embedded Evolutionary Distances). The xCEED methodology, which utilizes multidimensional scaling and Procrustes-related superimposition approaches, provides the ability to measure the global similarity between trees as well as incongruities between them. We demonstrate the application of this approach to the prediction of coevolving protein interactions and demonstrate its improved performance over the mirrortree, tol-mirrortree, phylogenetic vector projection, and partial correlation approaches. Furthermore, we show its applicability to both the detection of horizontal gene transfer events as well as its potential use in the prediction of interaction specificity between a pair of multigene families.

**Conclusions:**

These approaches provide additional tools for the study of phylogenetic trees and associated evolutionary processes. Source code is available at http://gomezlab.bme.unc.edu/tools.

## Background

Understanding historical relationships between genes, proteins and species is a core aspect of evolutionary biology, with the phylogenetic tree playing a fundamental role in analysis and visualization. However, major challenges still exist in the representation and analysis of the information encoded within phylogenetic trees. For instance, inferring the "true" tree is fundamentally a difficult problem, leading to continuous refinement of reconstruction methods [1]. Similarly, methodologies for tree comparison are also undergoing significant development [2]. In this instance, the typical goal is to compare trees in order to determine their degree of similarity, providing one mechanism to test a variety of hypotheses regarding evolutionary associations. For example, comparison of gene trees with organismal trees allows the detection of non-standard events such as horizontal gene transfer [3,4]. Comparison of species trees can be used to give a picture of host-parasite symbiosis as is seen, for example, in the case of attine ants, their fungal cultivars, and the Escovopsis parasite [5]. Another example is the prediction of protein-protein interactions, as it has been shown that interacting proteins often appear to coevolve with one another [6-8]. Such instances of coevolution are largely based on the premise that in order to maintain their interaction (and thus their broader functionality), changes in one gene/protein will be coordinated with changes in the other, and this process of coevolution or correlated evolution can be observed through the similarity of their phylogenetic trees [9,10].

While there are a variety of methods available for the comparison of trees, two general categories of approaches are clearly distinguishable. The first class of approaches focuses on comparing trees through topological features, for example quantifying the number of shared/non-shared substructures (e.g. subtrees of four leaf nodes) between a pair of trees [11,12] or finding the minimum number of operations (e.g. nearest neighbor interchange) to transform one tree into another [13-15]. The second class of approaches compares the distance or path length information directly. Specifically, in these approaches assessing the similarity between two trees is reduced to a problem of finding the degree of correlation (most commonly the Pearson correlation) between the elements within the respective distance matrices. The "mirrortree" method is based on such an approach and was developed for the prediction of protein-protein interactions [16]. Continued work in this area has led to multiple modifications of the basic mirrortree approach including the use of patristic distances obtained from the corresponding neighbor-joining tree instead of the observed inter-protein distances [17], the correction of patristic distance matrices for their inherent similarity due to background "tree of life" evolution [17-19], and the incorporation of ancestor node information into the distance matrices [20].

While methods based on distance matrix similarities have proven to be of particular value, several substantial disadvantages exist. For instance, these methods assume that each value in a distance matrix is independent of the other distance values. This is generally not the case as, if a distance (path length) between two leaf nodes changes, lengths of all other paths involving the modified edge(s) also change. Therefore, any method in which the distance matrices are directly manipulated without considering this dependency may bias the reported correlations. It is also difficult to extend these existing approaches, for example, to incorporate robust estimation into the identification of outlying lineages between compared trees. Furthermore, by definition, it is not possible to handle trees of different size or to align multiple trees simultaneously. Finally, prior knowledge cannot be readily incorporated so as to help guide comparisons.

Here, we report a novel method for the comparison of evolutionary distance matrices (and hence trees) based on the superimposition of Euclidean embeddings that best realize the given distance relationships. Specifically, we start from a set of aligned sequences and generate distance matrices based on either distance information calculated directly from the alignment, or distances derived from a corresponding neighbor joining tree. From these distance matrices we then map each sequence to a Euclidean space via metric multidimensional scaling (MDS). This operation produces a multidimensional structure or point pattern, where each point represents a taxon, and the distance relationships between all points is maintained from the original distance matrix. For the purpose of comparing two trees, the same operation is applied to the second distance matrix, generating the second Euclidean embedding. Finally, we superimpose one embedded point pattern onto the other with the degree of fit being determined by the least squares sum of deviations between corresponding point pairs or by some other measure as described below.

**Figure 1:**
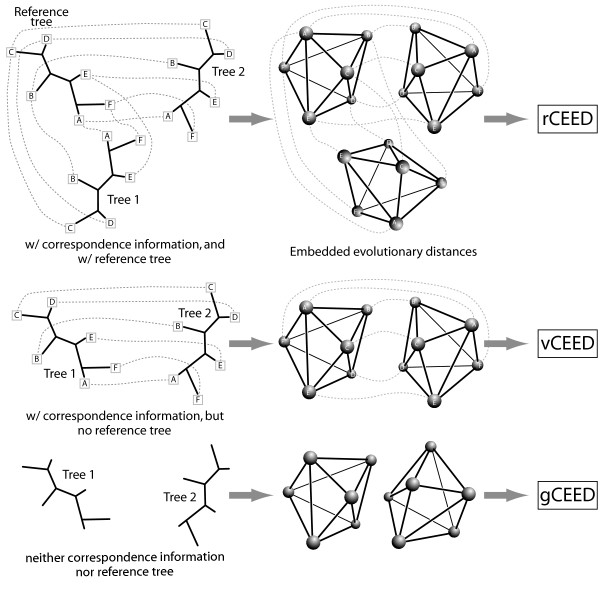
**The three different types of embedded structure alignment described in this work**. (a) rCEED aligns two target structures indirectly using a reference structure. This alignment is based on classical Procrustes superimposition. (b) For the detection of outliers and/or common substructures, we use vCEED to perform a local alignment (rather than global in the case of rCEED). (c) If neither a reference structure nor correspondence information is available, we can align the structures using gCEED which adapts a Gaussian mixture model approach for the accurate superimposition.

**Figure 2:**
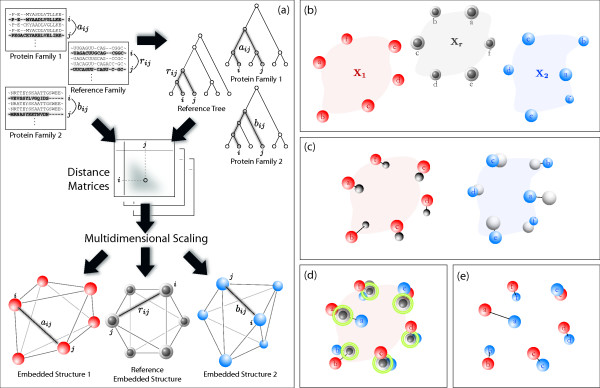
**Schematic overview of rCEED approach**. (a) Genetic distances obtained from sequence alignment or patristic distance obtained from phylogenetic tree are mapped into Euclidean space by multidimensional scaling. Orthologous protein families **X_1 _**and **X_2 _**along with two identical reference structures (16S rRNA orthologs), **X_r_**, are embedded in a Euclidean space. (b) Next, each reference structure is superimposed onto their respective protein families. (c) All four structures are now superimposed based on estimated transformations between each set of references. Since both reference structures were orthogonally transformed in (b), they will match exactly at this step. (d) The final superimposition result after removal of the reference structures.

In this paper, we refer to the general comparative approach of Euclidean embedding creation and alignment as "xCEED", the Comparison of Embedded Evolutionary Distances. However, this general approach actually contains three different superimposition methods, differing with regard to the question being asked or the data available (see Figure 1). Briefly, the first approach is an indirect superimposition of target structures (trees) that is guided by a low-noise reference structure, 16S ribosomal RNA phylogenies. While similar to the tol-mirrortree and vector-projection methods [17,18], this approach, rCEED, provides a new way to remove background correlation caused by tree-of-life evolution and thus helps in providing an accurate measure of coevolution (see Figure 2). Like the tol-mirrortree and vector-projection methods, rCEED requires both a reference structure as well as correspondence information for proper alignment (e.g. protein A in tree 1 maps to protein B in tree 2). We describe the application of rCEED to the prediction of coevolving protein interactions and demonstrate its improved performance over the mirrortree, tol-mirrortree [16,17], phylogenetic vector projection [18], and partial correlation methods [19].

In cases where the identification of incongruent region between trees is desired, robust structure alignment (vCEED) can be performed using "Verboonian" Procrustes [21], which penalizes less for the existence of outliers when compared to rCEED. As a result, one can detect local regions of similarity even in the presence of outliers and/or identify outliers relative to a common shared structure. The identification of horizontal gene transfer (HGT) events is an area where outlier detection within a phylogenetic tree is needed and we provide an example of the applicability of vCEED to this problem. As with rCEED, we can also use vCEED to detect coevolving protein interactions, especially in cases where a reference structure is not available and/or target structures (trees) contain outlying taxa and show its in this. We also compare the performance of vCEED with that of rCEED and other existing methods.

Finally, alignment without either a reference structure or mapping information can be performed with a Gaussian mixture model superimposition approach (gCEED). As a proof-of-concept for the potential broader utility of this approach, we describe its application to the prediction of protein interaction specificity between multigene families. As a whole, the xCEED methodology provides a novel approach to the tree comparison problem and the study of related evolutionary processes.

## Results and Discussion

### Prediction of protein interactions

We first applied both rCEED and vCEED to the prediction of protein interactions through the detection of a coevolutionary signal between orthologous protein families. While analogous to the approaches of [17,18], rCEED attempts to address some of their weaknesses. Specifically, in the tol-mirrortree approach, Pazos and colleagues subtracted the distance matrix of 16S rRNA from that of each protein, and then measured the correlation between these "difference of distance" matrices [17]. However, direct subtraction of rRNA from protein distances is problematic, as their evolutionary rates are different and it is not clear as to how to properly scale such differencing procedures. In phylogenetic vector projection, Sato and colleagues formed a vector from the lower triangular region of each distance matrix [18] and computed a difference vector between a gene vector and the same gene vector projected onto that of 16S rRNA. Again the correlation between distance matrices is measured with these difference (normalized) vectors. While avoiding direct subtraction of amino acid and rRNA distances, this approach (as does the tol-mirrortree approach) still assumes that all pairwise distances are independent. Not accounting for non-independence between distances can potentially cause bias in evaluation of correlation between two distance matrices [22].

The rCEED approach addresses these issues by viewing the leaf nodes in an embedded structure as independent variables. To measure the degree of coevolution, we estimate how similar the deviations from the reference structure are for each embedded structure. Doing this makes it possible to remove the background tree-of-life correlation without direct subtraction of rRNA distances from amino acid distances or assuming independence between distances. Specifically, we fit the reference structure(s) onto the first embedded structure and then onto the second structure separately (see Figure 2). Afterwords, we superimpose these two reference structures onto each other while carrying along their associated structures, which are the actual targets of interest. After this superimposition we can remove the reference structures, and then measure the degree of similarity between the remaining two target structures. As a single outlier can make the estimation of correlation coefficients unreliable [23] we also evaluated the use of vCEED in this application as it is specifically tailored for dealing with outliers (see following section as well as Methods for more details).

We compared the predictions of rCEED and vCEED to those of the mirrortree, tol-mirrortree, phylogenetic vector projection, and partial correlation methods using the data of Pazos and colleagues [17]. This data consisted of 388 protein interactions (true positives) out of a total of 19,972 possible between 188 *E. coli *proteins. Results are shown in Table 1 where we benchmarked the performance of all methods by computing the area under receiver operating characteristic curve (AUC) and estimated the significance by using the method of DeLong *et al*. [24]. We also provide the area under precision-recall curve, with the full precision-recall curves provided in additional file 1. As shown in Table 1, the AUC for the precision-recall curve was the greatest for vCEED with a value of 0.091, followed by rCEED using either patristic (0.083) or observed (0.069) distances. The worst performer was the mirrortree method with an PR-AUC of 0.048. Similar trends are observed when using the ROC score with rCEED having a score of 0.763, with that of mirrortree and tol-mirrortree being 0.687 and 0.722 respectively. The phylogenetic vector projection and partial correlation approach had ROC scores of 0.704 and 0.687 respectively. In all cases, the difference in AUC between rCEED and other methods was statistically significant (p-values ≈ 10^-6^). We also found that the AUC of vCEED was 0.763 - nearly that of rCEED using patristic distances.

**Table 1:** AUCs of tested approaches for detecting protein interactions via coevolution.

Methods	AUC (PR curve)^1^	AUC (ROC curve)^2^	p-value^3^
rCEED ^4^	0.069	0.763 ± 0.013	N/A
rCEED ^5^	0.083	**0.766 **± 0.012	0.7965
vCEED	**0.091**	0.763 ± 0.013	0.9919
mirrortree	0.048	0.687 ± 0.013	<0.0001
tol-mirrortree	0.063	0.722 ± 0.014	<0.0001
phylogenetic vector projection	0.053	0.704 ± 0.013	<0.0001
partial correlation	0.050	0.687 ± 0.013	<0.0001

Interactions identified in DIP^6^	388		

^1^Area under precision-recall curve

^2^Area under receiver operating characteristic curve

^3^The significance was computed using rCEED (observed distances) as reference according to [24].

^4^Based on observed distances

^5^Based on patristic distances after the reconstruction of neighbor joining trees

^6^August 2009 version of DIP.

### Detection of horizontal gene transfer

**Figure 3:**
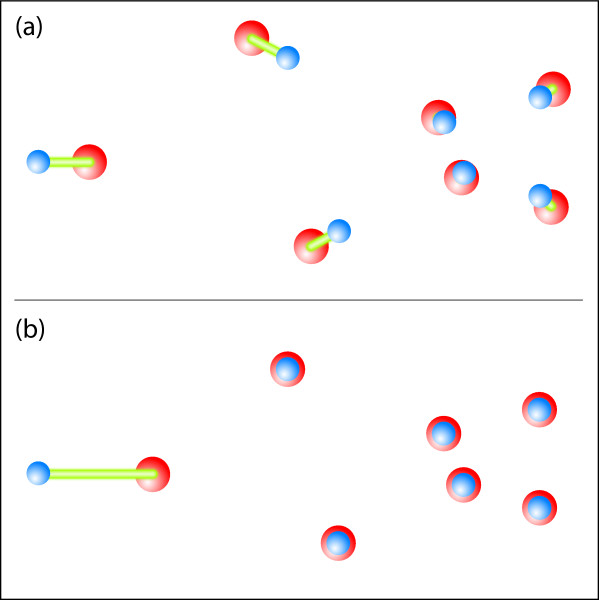
**Schematic of the difference between classical Procrustes alignment and Verboonian robust alignment (vCEED)**. Classical Procrustes alignment is shown in (a) with errors distributed across all corresponding pairs during global alignment. This is in contrast to vCEED (b), where an outlier becomes clearly distinguishable due to the alignment of a matching (local) substructure.

With the basic xCEED approach, we are able to estimate how well two trees match in a global sense through a least squares model. Specifically, if there exists an incongruent region between two trees, the least squares approach will tend to smooth away large local errors by allowing greater errors in other, otherwise well-aligning regions. However, in some cases we would prefer to maintain the best alignment of a substructure and/or be able to identify outliers that are not consistent with a comparison structure. To address this need, we adapted a robust Procrustes method previously proposed by Verboon and Heiser [21], with the difference between this and globally optimal superimposition diagrammed in Figure 3.

In Figure 3(a) it can be seen that errors are distributed across all pairs, as would be done using the basic xCEED method using least squares (e.g. rCEED with a reference structure). However, in this example there is a substructure that is in fact identical between the two that is lost as a result of the spreading of errors throughout the alignment. In contrast, Figure 3(b) shows the case where we have used Verboonian robust Procrustes (vCEED) for the alignment. In this case we have found and aligned the identical substructures; allowing identification of both this region of high-similarity as well as the outliers which deviate significantly between the two distance matrices.

This ability to detect local similarity and/or outliers is of particular utility in the identification of horizontal gene transfer (HGT) events. In HGT, a gene or group of genes is transferred laterally from another species, rather than inherited vertically from the parent(s). There are a variety of approaches to predict the occurrence of HGT based on, for example, codon usage, patterns of sequence homology, and patterns of gene distribution [25,26]. However, the most robust method for detecting HGT is through the comparison of phylogenetic trees of different genes. When a species accepts a gene laterally from another species, the location of the recipient species in the phylogenetic tree will be unusually close to the location of the donor species, which can be detected through manual analysis of the tree. Using vCEED, we can detect possible HGT by comparing a tree that potentially harbors one or more HGT events with a reference tree that does not, and then identifying the associated outliers as likely HGT candidates.

**Figure 4:**
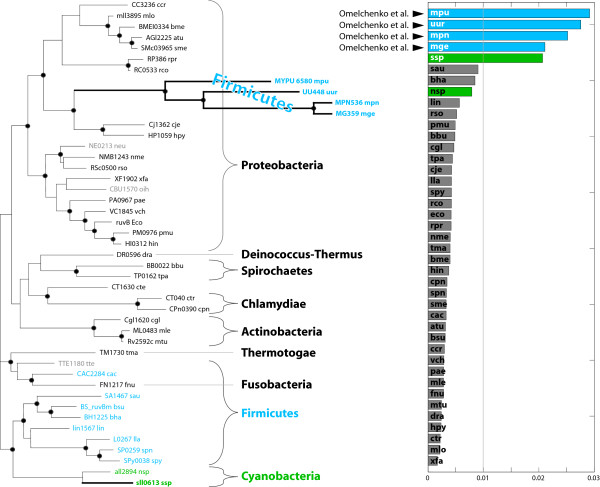
**HGT detection via vCEED for RuvB**. The phylogenetic tree of the *RuvB *(COG2255) family is shown on the left (redrawn from [27]). Shown on the right are the vCEED alignment errors between COG2255 and 16S rRNA. The vertical line at 0.01 was the threshold *c *we used in this analysis (see Equation (6)).

As a proof-of-concept, we applied vCEED to the case of the *RuvB *(COG2255) gene family described in [27]. In *E. coli*, the *RuvA *and *RuvB *proteins catalyze branch migration of Holliday junctions during genetic recombination and form an operon conserved in the majority of sequenced bacterial genomes. In contrast with the *RuvA *family, the *RuvB *gene is believed to have undergone HGT [27]. We compared the trees (as MDS-constructed embedding) of *RuvB *orthologous proteins collected from 41 bacterial species (see Methods) to that of 16S rRNA, with errors in the superimposition plotted in Figure 4. In this example, we expect that the lineages that underwent HGT will show up as outliers in the superimposition of the reference structure (16S rRNA) onto that of *RuvB*. As can be observed, genes with errors larger than the threshold of 0.01 for *c *(Equation (6), see Methods), in the superimposition are those from Ureaplasma and Mycoplasma and include *M. pulmonis *(MYPU_6570), *U. urealyticum *(UU449), *M. pneumoniae *(MPN535), and *M. genitalium *(MG358) (in blue). These four were the same species identified by Omelchenko and colleagues as being related to the HGT of the *RuvB *gene. In addition, vCEED was also able to identify sll0613, a Cyanobacterial gene from *Synechocystis *which, as can be observed in the phylogentic tree of *RuvB*, is closer to the Firmicutes rather than the Proteobacteria or Actinobacteria as opposed to *RuvA*.

**Figure 5:**
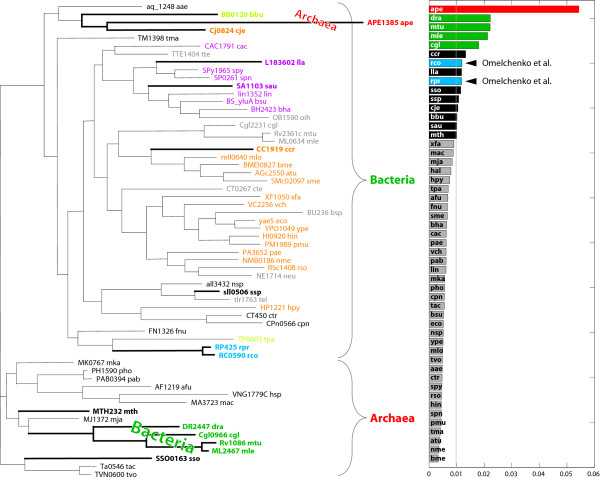
**HGT detection via vCEED for uppS**. The phylogenetic tree of the *UppS *(COG0020) family is shown on the left (redrawn from [27]). In addition to RP425 and RC0590 which was previously identified, an archaeal gene, APE1385, is clustered within a group of bacterial genes. Also observable is a bacterial branch consisting of DR2447, Cgl0966, Rv1086, and ML2467, with abnormal affinity to archaeal species. Both examples appear as outliers with vCEED (right) and indicate possible horizontal gene transfer. See Results for further details.

We also tested our approach with the more complicated case of the *UppS *gene family (COG0020) which, as also described in [27], is believed to harbor multiple HGT events. Figure 5 shows the outlying genes according to vCEED using 16S rRNA as the reference and using the same threshold value of 0.01 for *c *as in the previous example. As can be observed, we found that APE1385 from *A. pernix*, an archaeal gene, has the greatest divergence in the comparison to the 16S rRNA tree. We also see in the phylogenetic tree that it has atypical affinity to bacterial genes from *C. jejuni *(Cj0824) and *B. burgdorferi *(BB0120), both of which are also identified as weak outliers with errors just above threshold. Both Cj0824 and BB0120 would generally be expected to appear in the tree under the proper phyla, Proteobacteria (orange) and Spirochaetes (light green), respectively. Further examination of the identified outlier genes within the phylogenetic tree shows a bacterial branch (green) of *D. radioduran *(DR2447), *C. glutamicum *(Cgl0966), *M. tuberculosis H37Rv *(Rv1086) and *M. leprae *(ML2467), embedded within an archaeal phylum, the Euryarchaeota. We also see in the archaeal branch that a Crenarchaeota gene, SSO0163, stands out in its grouping with other genes from the Euryarchaeota phylum.

The Rickettsiales (blue) identified by Omelchenko and colleagues were also included in our outlier list, although they were not the most deviating. Note that being an outlier does not certify that the gene was horizontally transferred. Other mechanisms for this deviation can also occur including large differences in evolutionary rate or poor quality of the sequence alignment. Therefore, while this approach can potentially aid in the automatic prediction of potential HGT events, manual inspection of the phylogenetic tree may still be required. For example, the Firmicutes genes, L183602 and SA1103, while being slight outliers, are in a monophyletic subtree of Firmicutes (purple) and can thus be excluded from further consideration.

### Interaction specificity between multigene families

As demonstrated earlier, we can use either rCEED or vCEED to compare trees so as to predict the potential interaction of a pair of protein families. Again, these approaches require the use of mapping information to link the leaves of the two trees. There are applications, however, where one would like to compare trees that lack mapping information or where the recovery of mapping information is the primary goal. An important example of this type is in trying to determine likely interaction specificity between a pair of protein or domain families (e.g. receptor-ligand binding, etc.) [8,28-30].

Two primary methods for specificity prediction, MATRIX [28] and MORPH [29], currently exist, and like all methods, have their own inherent strengths and weaknesses. With MATRIX, a significant weakness is that the tree structure is completely ignored throughout the specificity search. MATRIX also requires multiple simulated annealing runs (≥ 100 runs with trees of 15 leaves or more) to determine which pairings are most frequent. Perhaps most important, both MATRIX and MORPH assume that there is a one-to-one correspondence between members of the two protein families; i.e. protein A from family 1 interacts solely with protein B from family 2. Thus it is not possible to generalize to the more realistic situation where we are looking at specificities between protein families of different size. In addition it precludes the possibility of many-to-many or multiple interaction partners for a given protein.

Here we adapt the use of a registration algorithm based upon Gaussian mixture models with our basic embedding and alignment approach [31]. In this case, we regard each vertex in the embedded structure (i.e. each leaf in the phylogenetic tree) as the mean of a Gaussian component such that the entire embedding is represented as a mixture model (see Methods). The central idea is that if we have two structures that are highly similar, as we align one structure closer to the other, their corresponding mixture models become accordingly similar. By trying to minimize the divergence between the two mixture models, we can eventually find the best superimposition. We refer to this method of alignment as Gaussian CEED or gCEED for short. Using gCEED, we attempted to determine the specificity information between protein families provided in Ramani *et al*. [28].

**Figure 6:**
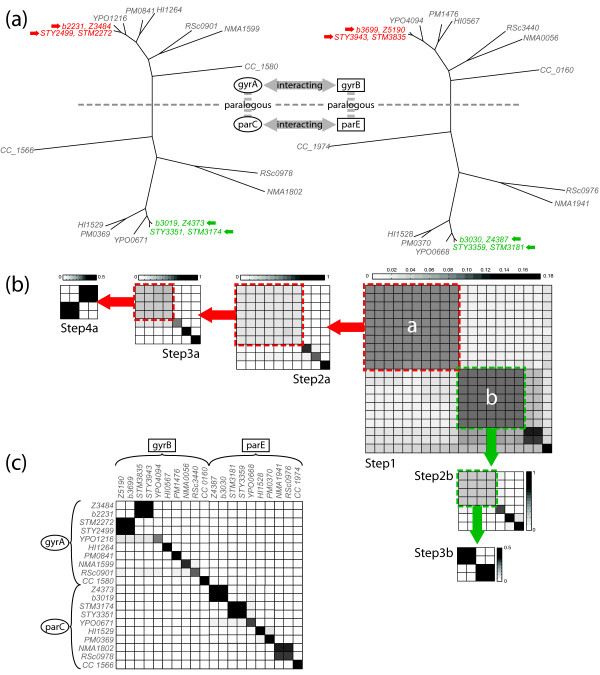
**Prediction of interaction specificity with gCEED**. (a) The phylogenetic trees and binding specificity between two multigene families, *GyrA/parC *and *GyrB/parE *(redrawn from [28]). (b) A series of probability matrices that visualize the recursive prediction of individual interaction specificities. Each colored box/arrow indicates the indeterminate block that was chosen for further alignment via gCEED. (c) The final probability matrix with predicted mappings in black/grey. A perfect prediction (assuming no cross-interactions) would be expected to show black squares along the diagonal and white squares everywhere else in the matrix.

The first example is the case of the interacting protein family of *GyrA *and *GyrB*. Each protein family is known to have a single paralog, *ParC *and *ParE *respectively, and these paralogs are also known to interact. Figure 6(a) shows the trees and interaction specificity (a leaf on one tree interacts with the corresponding leaf on the other tree) between these two multigene families. Results of the initial superimposition are shown in Figure 6(b)-Step1. The probability matrix is shown after having converted probabilities to grayscale values such that darker elements at [i, j] denote a higher probability of correspondence between *i*-th protein of family 1 and *j*-th protein of family 2. Proteins are arranged such that correct individual binding partners lie along the diagonal. In this first step we see that the initial alignment appears to have found the correct broader interaction specificity of *GyrA *with *GyrB *(region "a" in upper left of matrix) and *ParC *with *ParE *(region "b" and lower right) as observed by the distinct segmentation of the probability matrix into two distinct regions. For *ParC*/*ParE*, correct correspondence for three individual interactions was also found in the initial alignment (CC_1566 ⇔ CC_1974 as well as NMA1802 ⇔ NMA1941 and RSc0978 ⇔ RSc0976). Both regions *a *and *b*, being indeterminate, are separately superimposed in an iterative manner with results after each superimposition shown in the submatrices of Figure 6(b).

The final result after complete alignment is shown in Figure 6(c). Here we can see that gCEED successfully predicted the interaction specificity for 12 out of 20 individual interactions. The other misassigned 8 pairs were degenerate cases and their interaction specificity could not be further defined due to a lack of structural information. The reason for this can in part be observed within Figure 6(a), where the four proteins from each family (marked with arrows) can be observed to be very close to each other (short branch lengths from their common ancestor). In such instances it is difficult for the algorithm to find a correct high-probability mapping as multiple alignments are equally viable. Nevertheless, the interaction specificity at the protein-family level was correctly predicted. In addition, over half of the specific interactions could be recovered solely from the alignment of these structures.

We performed the same specificity analysis using gCEED to a total of 34 protein family pairs used in previous studies and compared results to that of MATRIX and MORPH in terms of stringent accuracy (Table 2). As can be observed, there is no significantly superior approach (Wilcoxon's signed rank test -data not shown), as all methods show instances where they have the greatest accuracy of specificity prediction. However, we emphasize the extra functionality of gCEED that is suited to realistic situations where (1) the size of the protein families at hand are unlikely to be identical, and/or (2) there exist some *a priori *knowledge of validated interacting protein interactions.

**Table 2:** Stringent accuracy of specificity prediction.

Protein Family Name	size	correlation	MATRIX	MORPH	gCEED
GyrA/B, ParC/E (*α*-proteobacteria)	20	0.9932	50.0	50.0	50.0
ParC/ParE (*α*-proteobacteria)	12	0.9921	50.0	66.7	66.7
Lyt-type regulator/sensors (E. coli/B. subtilis)	4	0.9709	50.0	50.0	50.0
GyrA/GyrB (Gram positive bacteria)	18	0.9795	33.3	44.4	55.5
Acetyl CoA carboxylase *α*/*β *(proteobacteria)	16	0.9756	75.0	75.0	62.5
ParC/ParE (bacteria)	26	0.9757	46.2	38.5	61.5
GyrA/GyrB (*α*-proteobacteria)	20	0.9723	90.0	80.0	50.0
ParC/ParE (Gram positive bacteria)	14	0.9634	14.3	28.6	28.6
CheA/CheB (bacteria)	8	0.9712	100.0	100.0	75.0
Pyruvate dehydrogenase *α*/*β *(bacteria)	17	0.9599	64.7	70.6	35.3
GyrA/B, ParC/E (Gram positive bacteria)	28	0.9484	10.7	7.1	10.7
DNA polymerase III E2/E3 (bacteria)	20	0.9378	20.0	40.0	70.0
Succinate CoA synthetase *α*/*β *(archaea)	13	0.9182	7.7	30.8	23.1
Ntr-type regulator/sensors (8 bacteria)	14	0.9025	28.6	42.9	21.4
Succinate CoA synthetase *α*/*β *(proteobacteria)	22	0.8959	54.6	50.0	54.5
Omp-type regulator/sensors (5 bacteria)	16	0.9307	0.0	68.8	31.3
CCR-type chemokine/receptor (mouse/human)	6	0.8790	66.7	66.7	33.3
Acetyl CoA carboxylase *α*/*β *(Gram positive bacteria)	9	0.8818	55.6	55.6	77.8
Chemokine/receptor (mouse/human/rat)	31	0.8789	19.4	16.1	3.2
CKR-type chemokine/receptor (mouse/human/rat)	18	0.8511	22.2	0.0	11.1
CheA/CheY (11 bacteria)	13	0.8370	23.1	15.4	23.1
Nar-type regulator/sensors (8 bacteria)	22	0.8488	18.2	9.1	13.6
GyrA/GyrB (archaea)	10	0.7948	20.0	20.0	10.0
Cit-type regulator/sensors (E. coli/B. subtilis)	5	0.7497	60.0	60.0	60.0
ABC transporter membrane/binding protein (E. coli)	17	0.4203	5.9	5.9	0.0
ABC transporter membrane protein 1/2 (E. coli)	19	0.6219	0.0	10.5	10.5
ABC transporter membrane binding protein (H. influenzae)	13	0.0427	15.4	23.1	7.7
Two-component sensor/regulators (E. coli)	27	0.6028	14.8	14.8	11.1
Chemokine/receptor (human)	13	0.5004	23.1	15.4	0.0
ABC transporter membrane protein 1/2 (H. influenzae)	14	0.3916	21.4	21.4	21.4
Omp-type regulator/sensors (E. coli/B. subtilis)	27	0.5314	7.4	33.3	3.7
Omp-type regulator/sensors (E. coli)	14	0.4295	28.6	14.3	14.3
Omp-type regulator/sensors (B. subtilis)	13	0.5628	15.4	7.7	15.4
Lyt, Ple, and other type regulator/sensors (8 bacteria)	20	0.4899	5.0	20.0	30.0

As a demonstration of this functionality within gCEED, we again used the case of *GyrA *and *GyrB *interactions. We first made the *GyrA *tree progressively smaller by sampling from nineteen down to ten sequences from the total of twenty *GyrA *orthologs, with 100 different combinations for each size. We then performed specificity prediction by aligning each sampled *GyrA *tree with the complete 20-node *GyrB *tree. To evaluate our performance, we introduce the *vicinity hit rate *as a means to estimate how close each node's true interacting partner is in relation to others within the aligned structures. Specifically, we define the vicinity hit rate as the ratio of nodes that have their true interacting parter within top three highest predicted probability partners. Thus the vicinity hit rate allows for situations where the true interacting partner is very close (but not the closest) to the predicted interaction partner as determined through the alignment.

**Figure 7:**
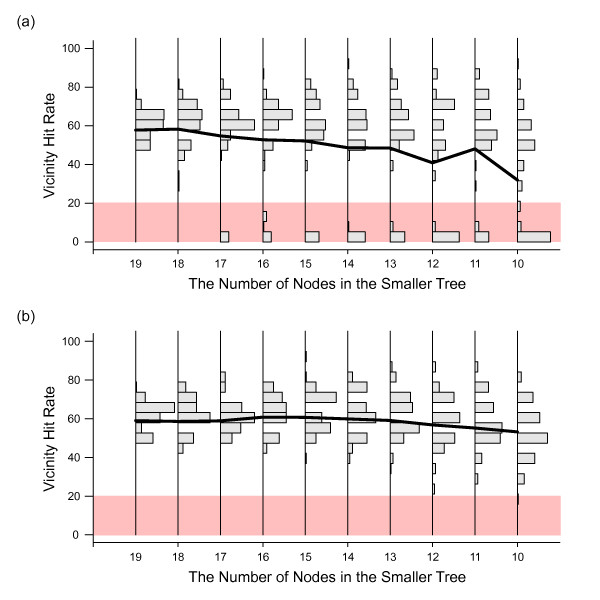
**Comparison of trees of different size**. The large tree is a 20-node *GyrB *tree. The smaller is a *GyrA *tree, formed from random sampling of nodes with sizes ranging from nineteen to ten nodes (x-axis). For each size of the smaller tree a histogram of vicinity hit rate is shown on the y-axis, based on 100 randomly-formed trees of a given size. The dark line specifies the average hit rate. (a) Accuracy of comparison without using any known interaction information. (b) Accuracy of comparison when using a single correct protein interaction pair as prior information.

Results of this analysis is shown in Figure 7(a). Again, each histogram along the x-axis was generated from 100 samples of the *GyrA *tree of corresponding size and the dark line shows how the average hit rate changes as the size of this tree decreases. In this instance, the ability for gCEED to determine binding specificities with a vicinity hit rate of approximately 65% (the hit rate generated in the original 20 vs. 20 superimposition) is relatively well maintained out to approximately 15 leaves or a 25% difference in tree sizes. As the difference between tree sizes decreases, we also begin to observe greater numbers of very poor predictions along with lesser numbers of very good predictions. These arise in situations where the the smaller tree fits very well, but in the wrong position within the larger tree, resulting in a very poor vicinity hit rate (shaded box in Figure 7(a)). The situation is analogous, but far less common for the high vicinity rate predictions (e.g. above 80%).

We would expect that additional information in the form of prior knowledge of an existing protein interaction pair would help to improve predictive performance. Such knowledge can be readily introduced into the gCEED alignment scheme and results of knowing just a single pair *a priori *are shown in Figure 7(b). Here we picked a random, but correct pair of interacting proteins between the two trees to serve as the *a priori *known information. As these proteins interact, we assume that they must be near each other in the final superimposition. We thus impose a constraint in the optimization of Equation (12), where the two proteins are kept within a pre-specified distance range (0.05 in this work).

Results show that use of prior knowledge provides a significant improvement in the stability of the vicinity hit rate, with a mean hit rate of approximately 60% even when reducing tree size to nearly half of its original value. In addition, using the structural information provided by the known interaction pair, we were able to avoid degenerate cases (shaded box in Figure 7(b)). In the comparisons between trees with greatest difference in size, the average vicinity hit rate of ten-node sample trees was 32.0% without prior knowledge versus 53.2% when using a single known protein pair. Together, these results suggest the potential for using gCEED in realistic situations where differences in tree sizes exist and/or prior information is available.

## Conclusions

In this work, we have described a novel approach for the comparison of phylogenetic trees, represented as embedded structures, and shown several examples of its application. First, when applied to the prediction of protein interactions, we see an improvement in prediction accuracy using the rCEED/vCEED approach when compared to other available approaches. We note, that high similarity between two embedded structures does not require that there is a physical interaction between members, but is only suggestive of the possibility. Similarly, the physical interaction between two proteins does not necessitate coevolution. Thus coevolutionary approaches such as those presented here can only identify a portion of the complete interactome within a given species. For the enhanced prediction of protein interactions, approaches such as rCEED/vCEED may show their greatest efficacy when combined with other computational approaches (e.g. [32-34]).

With vCEED, we were also able to perform a local alignment between structures, providing the opportunity to detect outliers that often indicate unusual evolutionary events including the horizontal gene transfer described here. While phylogenetic methods which detect incongruity between trees are generally considered the gold-standard for HGT detection, these methods are not readily automatable and require extensive manual analysis. Our results suggests that vCEED has significant potential in aiding such identifications.

By using the information inherent in the representation of a tree as an embedded structure, we were able to demonstrate the ability to align and measure the similarity between trees even when correspondence information is not available or when their sizes are different. While a basic example, the need to establish interaction specificity between interacting protein families supports the development of new approaches, and in this regard, gCEED shows significant promise.

While the embedding and superimposition of taxa within a Euclidean space in no way supersedes the use of a phylogenetic tree, it does provide several useful capabilities. For instance, embedding generates a deterministic structure that bypasses ambiguities associated in direct tree comparisons by transforming a specific distance matrix into a single specific shape enabling consistent comparison between trees. Similarly, use of a representative embedding also makes it possible to take into account the entire point-pattern structure all at once when determining correlation, rather than examining pair-by-pair correlation as in the mirrortree or related approaches. Finally, the representation of trees as embedded structures provides the capability to compare trees of different size, which is a built-in limitation of correlation-based methods. In this case, it becomes a matter of comparing two structures using procedures based on registration approaches such as the gCEED approach proposed in this work. As a whole, the xCEED approach provides an additional set of tools for the study of phylogenetic trees and associated evolutionary processes.

## Methods

### Data

For the prediction of protein interactions, we tested our method using data identical to that used by Pazos and colleagues [17]. This data set consists of experimentally characterized interactions among *Escherichia coli *proteins deposited in the February 2004 version of the DIP database [35]. For each protein in the interaction data, orthologs from 43 other prokaryotic species were collected to form each protein family. Among all the possible pairs of protein families, those that have less than ten common matching species (or taxa) were removed, leaving 19,972 suitable test protein interaction pairs (118 different proteins in total). From this complete set of protein interaction data, there were 115 experimentally characterized, true-positive, interaction pairs. We updated this set of interactions by checking all the 19,972 test interactions with the July 2007 version of DIP, and found that 388 of them were experimentally validated (an increase of 223 true-positive interactions from the 2004 version of DIP). We used this updated data set when measuring the discrimination power of our method. Along with this set of true interactions, a set of negative interactions was formed from the complement of this data - i.e. protein pairs not experimentally shown to be interacting. Thus a total of 19,584 negative interactions were formed in this way. For specificity prediction we used the data from [28].

Each protein family was aligned with clustalw[36], and distance matrices were calculated with the protdist routine from phylip[37]. These distance matrices are different from those used in [17] in that our data are created directly from the sequence alignments rather than from neighbor-joined trees. However, for comparison we also performed the same test with those used in [17]. The sequences and distance matrices of 16S rRNA were downloaded from the Ribosomal Database Project II [38].

### The basic xCEED approach: Classical MDS and superimposition with Procrustes

The approach we have developed is based upon extensions to the methods of multidimensional scaling and Procrustes analysis and we discuss these two fundamental approaches now. First, classical MDS attempts to find a Euclidean embedding of the data while simultaneously trying to preserve their interpoint distances [39]. Given distance matrix **D **= [*d*_*ij*_], we first compute the contrast matrix **M **which is defined to be equivalent to , where **C **is the centering matrix **I **- **1'1 **(**1 **is a row vector of ones and *n *is the number of nodes), and . After performing eigenvalue decomposition on **M**, which gives **M **= **QΛQ'**, we get **X **= **QΛ**^1/2^, which gives the coordinates of the points embedded in a, potentially high-dimensional, Euclidean space. Note that we truncate the negative eigenvalues in **Λ **since **D **is a Euclidean matrix if and only if **M **is positive semi-definite, which then defines the maximum dimensionality. Again, distances between points in this new structure representation are those that were provided by the original distance matrix for the tree.

Superimposition between two point sets of the same size, **W **and **Z**, is performed by Procrustes analysis. With Procrustes, we can superimpose point pattern **Z **onto point pattern **W **by applying *s *(dilation), **t **(translation), and **R **(rotation and reflection) to **Z**. Procrustes computes the optimal linear transformation, , such that *tr*((**W **- )(**W **- )') is minimized. Such minimum can be achieved when(1)

where **U **and **V **is the left and right singular matrices that are coming from the singular value decomposition of **Z**'**CW**(= **UΣV**'), where **Σ **is the matrix of singular values.

### Reference-based comparison of embedded evolutionary distances (rCEED): application to the quantification of protein coevolution

We first collect two sets of orthologous sequences from two potentially interacting protein families; respectively designated *F*_1 _and *F*_2_. In addition, we also assemble *F*_*r*_, which is a set of orthologous 16S rRNA sequences. Distance matrices, **D_1_**, **D_2_**, and **D_r_**, are then derived with respect to the species that are common to all *F*_1_, *F*_2_, and *F*_*r*_. The coordinates **X_1_**, **X_2_**, and **X_r_**, where each row represents the coordinate vector of a species embedded in Euclidean space, are produced from **D_1_**, **D_2_**, and **D_r _**by MDS. In cases where the dimensionality of the coordinate matrices are different, we zero-fill until the size of **X_1_**, **X_2_**, and **X_r _**are all minimally equivalent. We then find the robust superimposition between **X_1 _**and **X_2 _**by first superimposing **X_r _**onto both **X_1 _**and **X_2 _**independently(2)

such that *tr*((**X_1 _**- )(**X_1 _**- )') and *tr*((**X_2 _**- )(**X_2 _**- )') are minimized. Here  denotes the reference structure, **X_r_**, fitted to **X_i_**. Then we compute transformation parameters, *s*, **t**, and **R**, by superimposing  onto .(3)

Since both  and  represent the different orthogonal transformations of the same reference structure **X_r_**, this superimposition is an exact match. The final superimposition of **X_2 _**onto **X_1 _**is computed by simply applying to **X_2 _**the same parameters, , , and  obtained by (3).(4)

where  denotes **X_2 _**indirectly fitted onto **X_1_**. A schematic of our rCEED approach is given in Figure 2. Notice that we obtain a robust analytical solution for the superimposition parameters by putting the reference structure (in this case, **X_r _**and  in (2) and (3) always on the right hand side of the fitting equations. The standard root-mean-square deviation, *std. rmsd*, as a measure of structure similarity is given by:(5)

where  is the centroid of a reference structure. Because the number of common species will be different from one pair of protein families to another pair, their distributions in the space will have different variances. As a result, they are all normalized in (5), so that we can compare the strength of the coevolutionary signal among differently sized pair sets of protein families.

### Verboonian robust superimposition (vCEED): application to the detection of horizontal gene transfer

Verboon [21] proposed a robust method (Verboonian Procrustes) by adopting an alternative objective functions which put less penalty on errors over some threshold boundary. The direct consequence of this approach is that it brings us a better local alignment at the expense of allowing some outliers. Formally speaking, the transformation parameters are estimated by minimizing the loss function *L*(*s*, **R, t**) = ∑_*i*_*f*(*ε*_*i*_) where *ε*_*i *_is the residual distance between two corresponding points, and *f*(·) is a robust version of the error function. We adopted the Huber kernel [40] in this work,(6)

although other functions such as Lorentzian kernel or biweight function [41] are available. According to Verboon, we can minimize this loss function based on a weighted least squares model(7)

where **P **= [*p*_*ii*_] is a diagonal matrix of weight(8)

Since both transformation parameters (*s*, **R**, and **t**) and weight matrix (**P**) are unknown, we estimate them using Expectation-Maximization, where we alternate between the computation of transformation parameters using a fixed weight matrix **P **and the updating of **P **based upon the current estimation of transformation. Through this iterative process, the weight value in **P **gets smaller if an error term is larger than the pre-specified threshold, *c*. In the work described here, we used an empirically chosen value of 0.01 for *c*.

### Superimposition without correspondence information (gCEED): application to the prediction of interaction specificity

We adapted a registration algorithm based upon the Gaussian mixture model [31], where we regard each point in the point sets, **W **= {**w**_*i*_} and **Z **= {**z**_*j*_}, as the mean of each Gaussian component, . For this application we performed superimposition in 3-dimensional space due to the sparseness of the input tree data. Here we have two different mixture models,(9)

The central idea is that as we transform one point set closer to the other, the corresponding mixture models become similarly closer. We translate (**t**), rotate and project (**R**) the point set **Z **as before; the mixture model will then take the following form:(10)

Our goal then is to find the optimal **R **and **t **that minimize the dissimilarity between the two models *P*_*w *_and  using the *divergence D*.(11)

Both  and  are not a function of **R **and **t**. In addition, , because it is invariant with respect to **R **and **t**. Therefore, the minimization of (11) reduces to the problem of(12)

For the derivation of (12), see [42]. We assumed isotropy, so **Σ_i _**= **Σ_j _**= *σ*^2^**I **for all *i *and *j*'s. We further assumed that the weights of all Gaussian components are equal such that *α*_*i *_= 1/*m *and *β*_*j *_= 1/*n*.

## Authors' contributions

KC and SMG conceived the study, performed the research and analyzed the results. All authors wrote, read and approved the final manuscript.

## Supplementary Material

Additional file 1 **Precision-Recall curves for protein interaction predictions**. Precision-Recall curves for vCEED, rCEED (patristic distance), rCEED (observed distance), tol-mirrortree, phylogenetic vector projection, partial correlation, and mirrortree methods. The area under these Precision-Recall curves are shown in Table 1. Click here for file
