# Supplementary figures and images for: Comparison of phylogenetic trees through alignment of embedded evolutionary distances
Source: BMC Bioinformatics. 2009 Dec 15;10:423. doi: 10.1186/1471-2105-10-423 (PMC3087345; doi:10.1186/1471-2105-10-423)

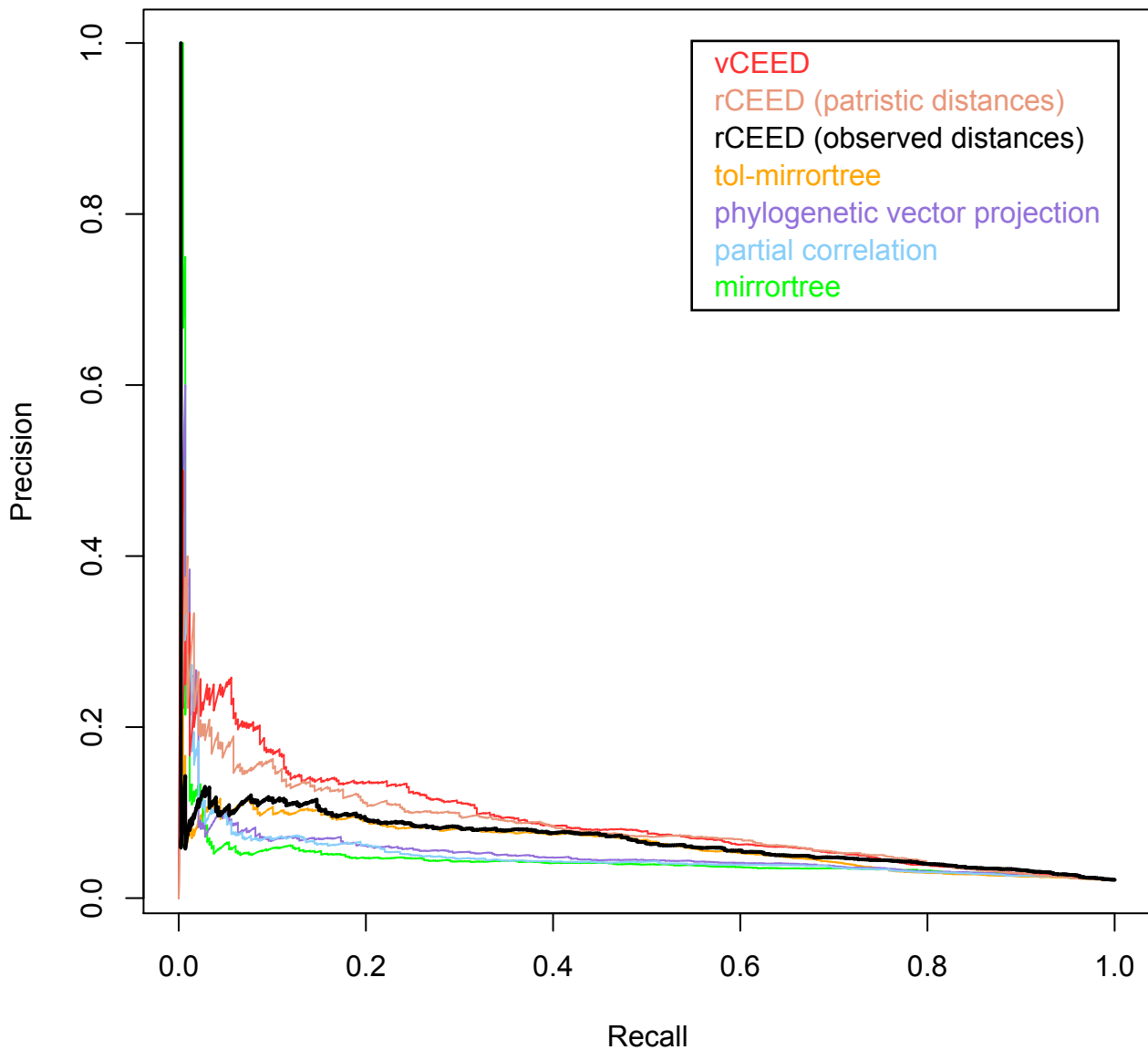

Supplement: Additional file 1 — Precision-Recall curves for protein interaction predictions. Precision-Recall curves for vCEED, rCEED (patristic distance), rCEED (observed distance), tol-mirrortree, phylogenetic vector projection, partial correlation, and mirrortree methods. The area under these Precision-Recall curves are shown in Table 1. [file 1471-2105-10-423-S1.PDF]
